# Efficacy of recombinant human soluble thrombomodulin for the treatment of acute exacerbation of idiopathic pulmonary fibrosis: a single arm, non-randomized prospective clinical trial

**DOI:** 10.1186/s40248-016-0074-z

**Published:** 2016-11-07

**Authors:** Sho Hayakawa, Yasuo Matsuzawa, Tamako Irie, Hagino Rikitake, Noriaki Okada, Yasuo Suzuki

**Affiliations:** Department of Internal Medicine, Toho University Medical Center, Sakura Hospital, 564-1 Shimoshidu, Sakura City, Chiba 285-8741 Japan

**Keywords:** Recombinant human soluble thrombomodulin, Acute exacerbation, Idiopathic pulmonary fibrosis

## Abstract

**Background:**

Coagulation abnormalities are involved in the pathogenesis of acute exacerbations of idiopathic pulmonary fibrosis (AE-IPF). The administration of recombinant human soluble thrombomodulin (rhTM), which has both anti-inflammatory and anticoagulant activities, improves outcomes and respiratory function in patients with acute respiratory distress syndrome. Therefore, we conducted a prospective clinical study to examine the effects of rhTM on respiratory function, coagulation markers, and outcomes for patients with AE-IPF.

**Methods:**

After registration of the protocol, the patients with AE-IPF who satisfied the study inclusion criteria were treated daily with 380 U/kg of rhTM for 7 days and steroid pulse therapy. The concomitant administration of immunosuppressants and polymyxin B-immobilized fiber column treatment was prohibited. The sample size was 10 subjects. The primary study outcome was the improvement of PaO_2_/FiO_2_ ratio a week after treatment initiation. Secondary outcomes were change in D-dimer level over time and 28-day survival rate in patients without intubation. Study data were compared with historical untreated comparison group, including 13 patients with AE-IPF who were treated without rhTM before the registration.

**Results:**

The mean PaO_2_/FiO_2_ ratio for the rhTM treatment group (*n* = 10) on day 8 significantly improved compared with that on day one (two-way analysis of variance, *p* = 0.01). The mean D-dimer level tended to decrease in the rhTM group on day 8, but the change was not significant. The 28-day survival rate was 50 % higher in the rhTM group than in the historical untreated comparison group, but the difference was not significant. A *post hoc* analysis showed that overall survival time was significantly longer in the treated group compared with that of the historical untreated comparison group (*p* = 0.04, log-rank test).

**Conclusion:**

rhTM plus steroid pulse therapy improves respiratory functions in patients with AE-IPF and is expected to improve overall patient survival without using other combination therapies.

**Trial registration:**

The study was registered with University Hospital Medical Information Network Clinical Trial Registry (UMIN-CTR) in October 2012 (UMIN000009082).

## Background

In patients with idiopathic pulmonary fibrosis (IPF), abnormal deposition of fibrin is found in the lung alveolar spaces [1], along with strong lung tissue expression of tissue factor, an initiator of blood coagulation, and plasminogen activator inhibitor-1 (PAI-1), an inhibitor of the fibrinolytic system [2]. Gunther et al. reported that the inhalation of heparin or urokinase suppresses the development of bleomycin-induced pneumonitis in rabbits [3], and Izuhara et al. reported that a PAI-1 inhibitor suppresses the development of bleomycin-induced pneumonitis in mice [4]. These findings suggested that anticoagulants can effectively treat IPF, but the efficacy of warfarin was not demonstrated when given to patients in the chronic phase of IPF [5].

Although IPF progression is usually gradual, acute exacerbation of IPF (AE-IPF) can also occur. Retrospective reviews have demonstrated that AE-IPF develops in 9.6 % of patients with IPF within 2 years of disease onset and that these patients have an extremely poor 3-month survival, which was found to be 40 % [6, 7]. Increased concentrations of circulating coagulation markers are also clinically observed in patients with AE-IPF [8]. Kubo et al. reported that the administration of low-molecular weight heparin improved the AE-IPF outcomes compared with those of untreated controls [9], but no subsequent reports have supported this finding.

Thrombomodulin is a membranous protein found on the surface of vascular endothelial cells. It directly inhibits clotting activity through the formation of a complex with thrombin and further suppresses blood coagulation through its activation of protein C. Thrombomodulin has anti-inflammatory as well as high mobility group box 1 (HMGB1) inhibitory and anticoagulant activities [10, 11]. Recombinant human soluble thrombomodulin (rhTM) demonstrates anticoagulation effects superior to heparin for the treatment of disseminated intravascular coagulation (DIC) [12] and improves the outcomes and respiratory functions in patients with sepsis-induced acute respiratory distress syndrome (ARDS) [13]. Furthermore, Ebina et al. reported reduced expression of thrombomodulin on the surface of vascular endothelial cells in autopsy cases of AE-IPF [14]. Based on these findings, we conducted a clinical study to examine the efficacy of rhTM treatment of AE-IPF.

## Methods

### Patients treated with rhTM

The study protocol was registered with the University Hospital Medical Information Network Clinical Trial Registry (UMIN-CTR) in October 10, 2012 (UMIN000009082). The study was a single center, single arm, non-randomized, prospective clinical trial, though we used the historical untreated comparison group. After registration, the patients who met all the following conditions were enrolled in this study: patients over 20 years old who were hospitalized in Sakura hospital, Toho-university medical center with a diagnosis of AE-IPF based on the diagnostic criteria, those who did not meet the exclusion criteria, and those who provided written informed consent.

The authors confirm that all related trials for this drug are registered. No incentives were presented for recruitment of the partecipants.

### IPF and AE-IPF diagnostic criteria

IPF was diagnosed according to the criteria of the American Thoracic Society/European Respiratory Society/Japanese Respiratory Society/Latin American Thoracic Association statement for IPF [15]. After the exclusion of pulmonary fibrosis due to collagen disease, professional exposure, hypersensitivity pneumonitis, and drug-induced lung disease, high-resolution computed tomography (CT; HRCT) images were evaluated by more than one respiratory specialist and experienced radiologists to confirm the usual interstitial pneumonia (UIP) pattern. Surgical biopsy was not performed.

AE-IPF was diagnosed according to the following criteria [6]: previous or concurrent IPF diagnosis; unpredicted acute worsening of dyspnea within 30 days; development of new ground-glass opacity or consolidation; and exclusion of pulmonary infection, left heart failure, pulmonary embolism, and acute lung injury from an already identified cause.

The following tests were performed to diagnose bacterial and viral infections: smear and culture tests of sputum, blood and urine that detect bacteria including acid-fast bacteria; antigen tests for influenza virus, *Mycoplasma pneumonia*, *Streptococcus pneumonia*, *Legionella pneumonia*, and cytomegalovirus; antibody test for *Mycoplasma pneumonia* and *Chlamydia pneumonia*; and assays for procalcitonin and β-D glucan. Patients were excluded if the possibility of infection could not be ruled out.

### Exclusion criteria

The patients who met any of the following conditions were excluded: 1) those with previous history of AE-IPF; 2) who was receiving corticosteroids or immunosuppressants; 3) who was receiving anti-coagulant therapy; 4) who had intracranial hemorrhage, pulmonary hemorrhage and/or gastrointestinal hemorrhage; 5) those with previous history of hypersensitivity against thrombomodulin; 6) those with active infectious disease; and 7) who was or possibly pregnant at the time of registration.

### Historical untreated comparison group

**Fig. 1:**
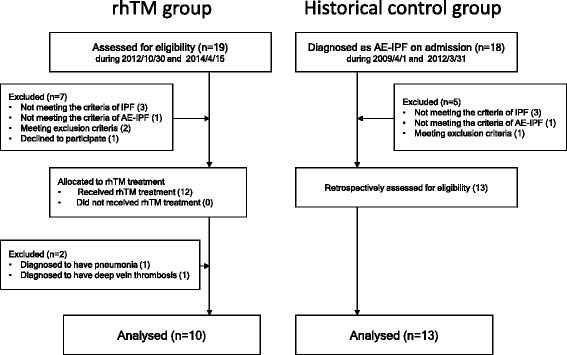
Flow diagram of the patients of two groups. rhTM: recombinant human soluble thrombomodulin

Out of 18 patients hospitalized in our department diagnosed as AE-IPF between April 2009 and May 2012, 13 were retrospectively assessed for eligibility based on the same criteria and exclusion criteria applied to the rhTM group. These 13 patients were enrolled in this study as “historical untreated comparison group (untreated group)” (Fig. 1).

### Medical treatment regimen

The rhTM group treatment regimen was as follows: intravenous infusion of methylprednisolone (m-PSL) at a dose of 1000 mg/day for 3 days (steroid pulse therapy) and then 80 mg/day for 7 days. The necessity and dose of steroid administration after this series was at the attending physician’s discretion. rhTM was dissolved in 100 mL of physiological saline and intravenously infused at a dose of 380 U/kg for 30 min, with initiation immediately after the start of steroid pulse therapy. Thereafter, rhTM was administered once a day for 7 days. All intervention was delivered at the general ward or intensive care unit of Toho university medical center, Sakura hospital by the ward nursing staff.

The concomitant administration of other anticoagulants, immunosuppressants, and elastase inhibitors, as well as hemoperfusion and polymyxin B-immobilized fiber column (PMX) treatment, was prohibited.

When oxygen saturation of 90 % could not be maintained with an FiO_2_ 50 % Venturi mask, noninvasive positive pressure ventilation (NPPV) was used. For NPPV, a BiPAP-Vision™ ventilator (Respironics Inc., Murrysville, USA), with which FiO_2_ could be accurately set, was used. However, intratracheal intubation was not actively recommended.

In the untreated group, all patients received steroid pulse therapy, and none was treated with rhTM. With regard to combination therapy, no restriction was applied in the untreated group.

### Serum markers

Serum level of Krebs von der lungen-6 (KL-6) and Surfactant protein D (SP-D) and HMGB1 were measured using an electrochemical luminescence immunoassay kit (EIDIA Co. Ltd., Tokyo, Japan; normal range < 500 U/ml), an enzyme immunoassay kit (Yamasa Corp., Chiba, Japan; normal range < 110 U/ml), and an enzyme-linked immunosorbent assay kit (Shino-test Corp., Tokyo, Japan), respectively. D-dimer (normal range < 0.5 μg/ml) and plasmin-α2 plasmin inhibitor complex (PIC) (normal range < 0.8 μg/ml) levels were measured using a latex photometric immunoassay kit (LSI Medience Corp., Tokyo, Japan), and thrombin–antithrombin complex (TAT) was measured using a chemiluminescent enzyme immunoassay kit (LSI Medience Corp., Tokyo, Japan; normal range < 3.0 ng/ml).

### Outcomes

The administration of rhTM for 1 week was reported to improve blood gas levels in patients with acute lung injury (ALI) [13]. In our historical untreated comparison group, the arterial oxygen partial pressure (PaO_2_)/FiO_2_ (P/F) ratio was not improved at 1 week after steroid pulse treatment. Therefore, P/F ratio improvement 1 week after starting treatment (day 8) was used as the primary study outcome. The P/F ratio was calculated from blood gas levels measured in the resting supine position with oxygenation at a flow rate of 12 L/min and FiO_2_ of 50 % using a Venturi mask. If endotracheal intubation or NPPV was performed, blood gas was measured using the FiO_2_ set at 50 % and the PEEP set at 0. In the first study protocol registered with UMIN-CTR, we set the secondary outcome as the P/F ratio on day 15 and day 29; but we excluded them later. The mean P/F ratio of untreated group on day 15 was higher than that on day 8 that is because two severe patients with poor P/F ratio had died before day 15. Comparison between the groups in P/F ratio on day 15 or 29 were thought to be inappropriate.

D-dimer concentrations for the 13 patients in the untreated group did not decrease during the treatment period (data not shown). Therefore, change in D-dimer concentration was assessed as a secondary outcome. Serum level of KL-6, SP-D, HMGB1, PIC and TAT were also evaluated.

The 28-day survival rate was also used as a secondary outcome. To avoid life extension due to intubation, the survival period was assessed only in patients without intubation.

### Target sample size

From the data obtained in the historical untreated comparison group, the standard deviation of the P/F ratio in patients with AE-IPF was presumed to be 50 to estimate the target sample size. In addition, the effect size was also assumed to be 1.0 for the change in the P/F ratio 1 week after the initiation of rhTM administration. Thus, the minimum target sample size was calculated to be 10 patients with a two-sided alpha level of 0.05 and a power of 80 %.

### Statistical analysis

The data were collected in Toho university medical center, Sakura hospital, and were statistically analyzed after linkable anonymizing of the data.

Unless otherwise noted, continuous variables were expressed as the mean ± standard deviation. Comparisons between two unpaired groups were performed using the Mann–Whitney *U* test. Differences in the P/F ratio on days 1 and 8 between the rhTM and untreated group were evaluated using two-way analysis of variance (ANOVA). Because the sphericity assumption was violated (Mauchly’s sphericity test, *p* < 0.0001), we used the Greenhouse–Geisser method. Differences in the longitudinal concentrations of D-dimer, TAT, PIC and HMGB1 in the rhTM group were tested using one-way repeated ANOVA. Differences in the study group 28-day survival rates and survival curves were compared using the chi square and log-rank tests, respectively. Statistical analyses were performed using SPSS 20.0 ((IBM® SPSS® Statistics). Probability values less than 0.05 were considered to be statistically significant.

## Results

### Patients' characteristics

A patient flow diagram is shown in Fig. 1. The date range for participant recruitment was from 2012/10/30 to 2014/4/15, and follow up period was until 2014/9/30. 19 patients were assessed for eligibility, 6 patients were excluded based on the aforementioned inclusion and exclusion criteria. One patient refused to participate in the study. So, rhTM administration was initiated in 11 patients. Another patient was excluded from the analysis after completion of rhTM administration, because he was turned out to have immunodeficiency and have pneumocystis pneumonia. Thus, the remaining 10 patients comprised the rhTM group (Fig. 1).

Table 1 shows patients' background characteristics for the rhTM group and historical untreated comparison group. Background characteristics of the two groups were generally similar.

**Table 1:** Patients' background characteristics

	rhTM group (*n* = 10)	Untreated group (*n* = 13)	*p*
Baseline characteristics			
Male, *n*	8	11	0.8
Age	73.2 ± 9.5	69.7 ± 8.5	0.3
Pulmonary function prior to acute exacerbation			
FVC (ml)	2200 ± 910 (*n* = 8)	1630 ± 570 (*n* = 7)	0.1
%FVC (% predicted)	68.1 ± 24.1 (*n* = 8)	58.6 ± 16.7 (*n* = 7)	0.3
PaO_2_ (mmHg)	68.9 ± 6.5 (*n* = 6)	64.4 ± 10.2 (*n* = 8)	0.4
Treatment of IPF prior to acute exacerbation			
Pirfenidone	2/10	0/13	
Steroids	0/10	0/13	
Immunosuppressant (cyclosporine)	0/10	0/13	
Anticoagulant therapy	0/10	0/13	
Home oxygen therapy	2/10	2/13	

Data are expressed as the mean ± standard deviation

*p*: rhTM group vs.untreated group

*rhTM* recombinant human soluble thrombomodulin, *FVC* forced vital capacity, *IPF* idiopathic pulmonary fibrosis

Table 2 reports baseline data obtained just before treatment initiation for AE-IPF. There were no significant differences between the two groups at this time point.

**Table 2:** Patients' parameters immediately prior to treatment initiation

	rhTM group (*n* = 10)	Untreated group (*n* = 13)	Normal range	*p*
PaO_2_/FiO_2_ ratio	168 ± 56	183 ± 47	N.A	0.5
CRP (mg/dl)	11.5 ± 8.3	11.0 ± 11.1	<0.03	0.6
LDH (IU/l)	378 ± 118	444 ± 173	120–240	0.4
KL-6 (U/ml)	1512 ± 583	2060 ± 1520	<500	0.8
SP-D (ng/ml)	482 ± 527	676 ± 711	<110	0.2
D-dimer (μg/ml)	5.3 ± 7.9	6.9 ± 8.9	<0.5	0.5
TAT (ng/ml)	8.1 ± 13.5	N.D	<3.0	
PIC (μg/ml)	3.0 ± 5.1	N.D	<0.8	
PAI-1 (ng/ml)	59.0 ± 52.4	N.D	≦50	
ProteinC (%)	84.7 ± 25.1	N.D	64–135	
ATIII (%)	90.9 ± 15.1	N.D	83 × 128	
HMGB1 (ng/ml)	10.6 ± 4.7	N.D	N.A	
Thrombomodulin (U/ml)	16.1 ± 5.9	N.D	12.1–24.9	

Data are expressed as the mean ± standard deviation

*p*: rhTM group vs. untreated group

*N.D*. no data, *N.A*. not available

*AE-IPF* acute exacerbation of idiopathic pulmonary fibrosis, *rhTM* recombinant human soluble thrombomodulin, *CRP* C-reactive protein, *LDH* lactate dehydrogenase, *KL-6* Krebs von der lungen-6, *SP-D* surfactant protein D, *TAT* thrombin–antithrombin complex, *PIC* plasmin-α2 plasmin inhibitor complex, *PAI-1* plasminogen activator inhibitor-1, *AT III* antithrombin III, *HMGB1* high mobility group box 1

### Therapeutic interventions for AE-IPF

All patients in the untreated group simultaneously received empirical administration of antibiotics with steroid pulse therapy, but only 4 out of the 10 patients in the rhTM group were treated with antibiotics either before or after rhTM administration. In the untreated group, 6, 6, and 3 patients received an elastase inhibitor (sivelestat sodium), an immunosuppressant (cyclosporine or cyclophosphamide), and PMX treatment, respectively. One patient received elastase inhibitors and one received immunosuppressants. Five patients received both. Three of these five patients received triple therapy, including PMX. No patients in the rhTM treatment group received these treatments.

Four out of the 13 patients in the untreated group underwent endotracheal intubation. In the rhTM group, 1 patient required endotracheal intubation and another patient was provided with NPPV (Table 3).

**Table 3:** Therapeutic interventions for AE-IPF

	rhTM group (*n* = 10)	Untreated group (*n* = 13)
m-PSL pulse therapy	10/10	13/13
rhTM therapy	10/10	0/13
Empiric antibiotic therapy	4/10	13/13
Sivelestat sodium hydrate	0/10	6/13
Immunosuppressant (cyclosporine, cyclophosphamide)	0/10	6/13
Polymyxin B-immobilized fiber column treatment	0/10	3/13
Noninvasive positive pressure ventilation	1/10	0/13
Invasive mechanical ventilation	1/10	4/13

*AE-IPF* acute exacerbation of idiopathic pulmonary fibrosis, *rhTM* recombinant human soluble thrombomodulin, *m-PSL pulse therapy* intravenous methylprednisolone 1 g/day for 3 days, *rhTM therapy* intravenous recombinant human soluble thrombomodulin of 380 U/kg/day for 7 days

### Changes in the P/F ratio 1 week after treatment initiation

The P/F ratios at the start of therapy (day 1) and on day 8 of therapy were 168 ± 56 and 215 ± 88, respectively, in the rhTM group, and 183 ± 47 and 157 ± 55, respectively, in the historical untreated comparison group. There was a significant statistical interaction between group and time point (Greenhouse–Geisser method, *p* = 0.005). Further analysis of the simple main effect revealed that the mean P/F ratio for the rhTM group on day 8 was significantly higher than that on day 1, although it was not significantly different from the untreated group value on day 8 (two-way ANOVA: *p* = 0.01 and *p* = 0.07, respectively) (Table 4).

**Table 4:** Change in P/F ratio one week after treatment initiation

		Day 1	Day 8
P/F ratio	rhTM group	168 ± 56*^,^**	215 ± 88*^,^***
	Untreated group	183 ± 47**	157 ± 55***

Data are expressed as the mean ± standard deviation

*P/F ratio* PaO_2_/FiO_2_ ratio

*rhTM* recombinant human soluble thrombomodulin

*The mean P/F ratio of the rhTM group on day8 was significantly higher than on day1 (two-way ANOVA, *p* = 0.01)

**The difference between the group mean P/F ratios on day 1 was not significant (two-way ANOVA, *p* = 0.5)

***The difference between the group mean P/F ratios on day 8 was not significant (two-way ANOVA, *p* = 0.07)

### rhTM group changes in coagulation markers and HMGB1

**Fig. 2:**
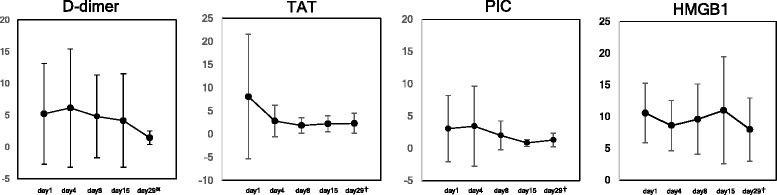
Serial changes in coagulation markers and HMGB1 levels of the patients in the rhTM group (*n* = 10). There were no significant changes between the serial change in D-dimer, TAT, PIC and HMGB1 (one-way repeated ANOVA). Data are expressed as the group means ± standard deviation. HMGB1: high mobility group box 1; TAT: thrombin-antithrombin complex; PIC: plasmin-α2 plasmin inhibitor complex. *n = 7, †*n* = 5

In the rhTM group, D-dimer as well as TAT and PIC concentrations tended to decrease, but the changes were not statistically significant. No significant changes were noted in HMGB1 values from the initiation of the treatment to 28 days’ post-treatment (Fig. 2).

### Survival rate

**Fig. 3:**
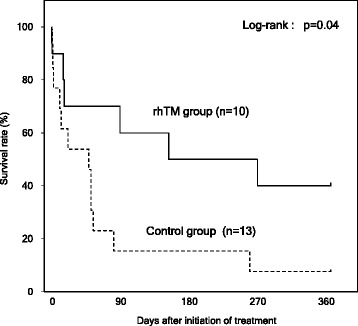
Kaplan–Meier distribution for the probability of survival without tracheal intubation. The *solid line* represents patients in the recombinant human soluble thrombomodulin (rhTM) group, and the *dotted line* represents patients in the untreated group. Survival was significantly better in the rhTM group than in the untreated group at 1 year (*p* = 0.04, log-rank test). rhTM: recombinant human soluble thrombomodulin

The survival rate for the rhTM group 28 days after the initiation of treatment was 70 %, which was higher than that in the historical untreated comparison group (54 %), although the difference was not significant. A *post hoc* analysis revealed that survival rates for the rhTM and untreated groups were 70 and 23 % at 2 months, 60 and 15 % at 3 months, and 40 and 8 % at 12 months, respectively, after treatment initiation. The median survival times for the rhTM and untreated groups were 153 and 48 days, respectively. At 1 year, the rhTM group had a significantly better survival outcome than the untreated group (*p* = 0.04 by the log-rank test) (Fig. 3).

### Safety

Adverse events attributable to rhTM such as hemorrhage were not reported.

## Discussion

The present study demonstrated that combined rhTM and steroid therapy improved the P/F ratio measured 1 week after treatment initiation without any concomitant use of cyclosporine or PMX and produced significantly longer patients' survival than patients under historical treatment. Thus, rhTM appears to be effective in the clinical treatment of AE-IPF.

Recently, retrospective studies on the effects of rhTM on AE-IPF were reported from 3 institutions in Japan, with all studies reporting favorable effects for rhTM. Tsushima et al. reported that the 28-day survival rate was 65 % in 20 patients treated with rhTM and 17 % in 6 patients who were not treated with rhTM [16]. Likewise, Isshiki et al. reported that the 90-day survival rates were 69 and 40 % in 16 patients treated with rhTM and 25 untreated patients, respectively [17], while Kataoka et al. reported that the 90-day survival rates were 70 and 35 % in 20 patients treated with rhTM and in 20 control patients, respectively [18].

There are some differences between the present study and the 3 aforementioned studies. First, the sample size in our study (10 patients in the rhTM group and 13 in the untreated group) was smaller than that in the 3 previously reported studies. Furthermore, while the 3 studies were conducted in a retrospective manner, our study was conducted in a prospective manner according to a previously published protocol (UMIN 000009082) that included all patients with AE-IPF who provided informed consent and who were registered after October 2012. In addition, there were some differences between studies in the treatment methodologies utilized. Although all studies, including ours, used steroid pulse therapy and subsequent steroid administration, Kataoka et al. concomitantly administered cyclosporine to all 20 patients, and Isshiki et al. administered cyclosporine to 15 of the 16 patients in their studies. The effectiveness of cyclosporine for AE-IPF has already been demonstrated [19, 20], and we also concomitantly used it to treat 6 of the 13 patients in our untreated group. However, we prohibited the concomitant use of cyclosporine for the following reasons: cyclosporine administration has not been accepted as a standard therapy for AE-IPF yet. There are concerns regarding its considerable cost and adverse reactions, and our goal was to assess the efficacy of rhTM alone. The concomitant use of sivelestat sodium and PMX was also prohibited in the rhTM group, although these drugs were used to treat some patients in the untreated group (Table 3).

It has been reported that NPPV shows certain efficacy in AE-IPF [15, 21], but some investigators are against the use of invasive mechanical ventilation. In our untreated group, none of the patients used NPPV, while 4 out of the 13 patients underwent endotracheal intubation, all of whom died without being weaned from it. We adopted a policy to not actively introduce endotracheal intubation in the patients treated with rhTM, although we introduced NPPV when the use of a Venturi mask could not maintain an oxygen saturation of 90 % at FiO_2_ of 50 %. In addition, survival time was calculated using data obtained from patients without endotracheal intubation to avoid the potential influence of the life-extending effects of endotracheal intubation on study results. In the rhTM group, 1 patient each who underwent NPPV and endotracheal intubation died within 28 days of the initiation of rhTM therapy.

Collard et al. compared biomarker profiles between patients with ALI and those with AE-IPF and reported that protein C levels significantly decreased in the former group but that levels remained within the normal range in the latter group [8]. In the rhTM group patients in the present study, protein C levels were also within the normal range before initiating rhTM treatment (Table 2). The concentrations of D-dimer, TAT, and PIC were increased before rhTM treatment, but all increases were moderate, and only 2 patients met the Japanese diagnostic criteria for acute DIC [22]. The concentrations of D-dimer, TAT, and PIC tended to decrease with rhTM treatment, but the differences were not statistically significant (Fig. 1).

On the other hand, Kataoka et al. reported that D-dimer concentrations in bronchoalveolar lavage (BAL) fluid were close to those in serum for patients with AE-IPF [18]. Because BAL fluid is a dilution of the alveolar lining fluid, the D-dimer concentration in the alveolar lining fluid was considerably higher than that in serum in their study. Therefore, it is speculated that greater than expected coagulation abnormalities occur in regional lung tissues in AE-IPF. Thus, it may be better to evaluate the concentrations of coagulation markers in BAL fluid than in serum.

In the present study, the baseline concentration of serum thrombomodulin did not exceed the normal range in the 10 patients in the rhTM group (Table 2). Collard et al. reported that serum thrombomodulin concentrations were lower in patients with AE-IPF than in those with ALI and suggested that low thrombomodulin is a poor prognostic factor [8]. In addition, Ebina et al. reported a decrease in thrombomodulin expression in autopsied tissue from patients with AE-IPF [14]. Furthermore, Ware et al. reported that thrombomodulin concentration in pulmonary edema fluid was higher than that in the serum of patients with ALI/ARDS [23]. On the other hand, Kataoka et al. reported that the concentration of thrombomodulin in BAL fluid was 10-fold lower than that in serum in patients with AE-IPF. The same study also showed that the D-dimer concentration in BAL fluid was nearly equal to that in serum [18]. Therefore, it is assumed that the thrombomodulin concentration is relatively lower than the D-dimer concentration in the alveolar space. These findings suggest that there is a relative deficiency of thrombomodulin in the lung tissue and serum of patients with AE-IPF; thus, supplementation with rhTM in these patients should improve their condition.

Regarding the mechanism of action of rhTM, it was reported to lower blood HMGB1 concentration. Ebina et al. reported that the HMGB1 concentration in BAL fluid increases in a time-dependent manner in patients with AE-IPF [14]. Tsushima et al. also reported that the serum HMGB1 concentration increases in a poor outcome group but is unchanged in a good outcome group [16]. However, marked changes in HMGB1 concentration were not observed in any patient in our study (Fig. 2). It is necessary to clarify the time course change in HMGB1 concentration in BAL fluid to verify whether rhTM induces improvements in disease outcomes through its regulation of HMGB1.

There are some limitations to this study. First, the study was a single center, single arm, non-randomized clinical study, though we used the historical untreated comparison group. Second, the sample size was small, although significant improvement was demonstrated even with this small cohort, suggesting the excellent efficacy of rhTM. There were, however, large variations in patients' background factors before acute exacerbation, the data obtained during acute exacerbation, and the changes in P/F ratio after rhTM administration; clinical courses substantially differed among patients. A larger scale study is required to assess the efficacy of rhTM for treating various types of patients. Third, we did not examine BAL fluid to diagnose AE-IPF. The main purpose of BAL fluid examination was to eliminate the possibility of an active infection. Instead of examining BAL fluid, we determined the presence or absence of an infectious disease by clinical examination, culture tests, antigen testing, antibody testing, and procalcitonin level measurement. An antibacterial agent was administered prior to rhTM treatment when infectious disease was suspected, and rhTM treatment was started only when the absence of an infection was confirmed. Antibacterial agents were given to 3 patients whose symptoms did not improve after starting rhTM plus steroid treatment, but their symptoms also did not improve with antibacterial administration, and they subsequently died. Antibacterial agents were not administered to the remaining 7 patients until they were discharged. The possibility of an opportunistic infection was low in the 10 rhTM subjects included in the analysis because they did not receive any steroid or immunosuppressant treatment and did not have poorly controlled diabetes. Therefore, the possible involvement of a known infectious disease was considered to be low in the disease conditions of the patients with AE-IPF who received rhTM treatment. However, as mentioned above, BAL fluid examination is thought to be useful to assess coagulation and fibrinolytic functions as well as to eliminate the possibility of infection. Thus, examination of BAL fluid as well as measurement of coagulation and fibrinolytic markers in BAL fluid should be performed when patient's conditions permit.

## Conclusions

In conclusion, our findings showed that treatment with rhTM and a steroid improves oxygenation in patients with AE-IPF and is expected to improve their survival without the use of other combination therapies. Larger scale controlled trials are necessary to further demonstrate the efficacy of rhTM in future patient populations.

## Abbreviations

AE-IPF: Acute exacerbation of idiopathic pulmonary fibrosis
ARDS: Acute respiratory distress syndrome
DIC: Disseminated intravascular coagulation
HMGB1: High mobility group box 1
IPF: Idiopathic pulmonary fibrosis
KL-6: Krebs von der lungen-6
m-PSL: Methylprednisolone
NPPV: Noninvasive positive pressure ventilation
P/F ratio: PaO_2_/FiO_2_ ratio
PAI-1: Plasminogen activator inhibitor-1
PIC: Plasmin-α2 plasmin inhibitor complex
PMX: Polymyxin B-immobilized fiber column
rhTM: Recombinant human soluble thrombomodulin
SP-D: Surfactant protein D
TAT: Thrombin–antithrombin complex
UIP: Usual interstitial pneumonia
UMIN-CTR: University hospital medical information network clinical trial registry
